# Association Between Stress Testing–Induced Myocardial Ischemia and Clinical Events in Patients With Multivessel Coronary Artery Disease

**DOI:** 10.1001/jamainternmed.2019.2227

**Published:** 2019-07-22

**Authors:** Cibele Larrosa Garzillo, Whady Hueb, Bernard Gersh, Paulo Cury Rezende, Eduardo Gomes Lima, Desiderio Favarato, José Antônio Franchini Ramires, Roberto Kalil Filho

**Affiliations:** 1 Instituto do Coração (InCor), Hospital das Clínicas da Faculdade de Medicina da Universidade de São Paulo, São Paulo, Brazil; 2 Department of Medicine, Mayo Clinic College of Medicine, Rochester, Minnesota

## Abstract

**Importance:**

The long-term prognostic implications of myocardial ischemia documented during stress testing in patients with multivessel coronary artery disease (CAD) are unclear.

**Objective:**

To assess whether documented stress testing–induced myocardial ischemia is associated with major adverse cardiovascular events or ventricular function changes in patients with stable multivessel CAD.

**Design, Setting, and Participants:**

A prospective cohort study was conducted using data from a single-center randomized clinical trial (Medicine, Angioplasty, or Surgery Study [MASS] II) to examine the association of myocardial ischemia documented during stress testing at baseline with cardiovascular events and ventricular function changes during follow-up. Participants were previously randomized (May 1, 1995, to May 31, 2000) to medical therapy, percutaneous coronary intervention with bare metal stents, or coronary artery bypass grafting. Event-free survival was estimated by the Kaplan-Meier method, and multivariable Cox regression models were calculated to assess the association between ischemia and the primary composite end point. The vital status was determined on February 28, 2011. Data were analyzed from February 1, 2016, to April 1, 2017.

**Main Outcomes and Measures:**

Cardiovascular events (overall mortality, myocardial infarction, and revascularization for refractory angina) were tracked from the time of randomization to the end of the 10-year follow-up (mean [SD] duration, 11.4 [4.3] years). Myocardial ischemia was assessed at baseline and at 1-year intervals by exercise stress testing, and ventricular function (left ventricular ejection fraction) was assessed by echocardiography at baseline and after 10 years. Patients with documented ischemia were compared with those without ischemia regarding the outcomes and changes in ventricular function.

**Results:**

Of 611 participants, 535 underwent exercise stress testing at baseline: 270 with documented ischemia and 265 without. Of these 535 patients, 373 (69.7%) were men, and the mean (SD) age for the entire cohort was 59.7 (9.2) years. No association was found between the presence of ischemia at baseline and survival free of combined cardiovascular events (hazard ratio, 1.00; 95% CI, 0.80-1.27; *P* = .95) after multivariable adjustment that included CAD initial randomized treatments. In addition, among 320 patients who underwent echocardiographic evaluation, the slight decline in left ventricular ejection fraction after 10 years was similar in both groups (median [SD] difference, −4.9% [18.7%] vs −6.6% [20.0%], respectively, for groups with and without ischemia; *P* = .97).

**Conclusions and Relevance:**

In this study, regardless of the therapeutic strategy applied, the presence of documented myocardial ischemia did not appear to be associated with an increased occurrence of major adverse cardiovascular events or changes in ventricular function in patients with multivessel CAD during a long-term follow-up.

## Key Points

**Question:**

Is stress testing–induced myocardial ischemia associated with major adverse cardiovascular events or ventricular function changes in patients with multivessel coronary artery disease?

**Findings:**

In this cohort study using 10-year follow-up data from the Medicine, Angioplasty, or Surgery Study (MASS) II randomized clinical trial, 270 patients with stress-induced myocardial ischemia showed similar rates of major adverse cardiovascular events and ventricular function changes compared with 265 patients without stress-induced ischemia.

**Meaning:**

The presence of myocardial ischemia in patients with stable multivessel coronary artery disease neither increases risk for major adverse cardiovascular events nor identifies patients whose condition will evolve with ventricular function changes in a long-term follow-up.

## Teaser

This cohort study uses MASS II trial data to assess the association of myocardial ischemia documented during exercise stress testing with major adverse cardiovascular events or changes in the ventricular function in patients with multivessel coronary artery disease.

## Introduction

Previous studies^1,2^ suggested that the presence of myocardial ischemia during stress testing and ambulatory electrocardiographic monitoring indicated an increased risk of cardiac events. Some studies also suggested that the severity and extent of abnormalities demonstrated during resting myocardial perfusion single-photon emission computed tomography as well as the amount of stress-induced ischemia were associated with adverse cardiovascular outcomes.^3^ Consequently, the severity of ischemia is generally accepted to be one of the indications for revascularization.^4^

However, contemporary data from the Clinical Outcomes Utilizing Revascularization and Aggressive Drug Evaluation (COURAGE) trial substudy^5^ indicated that the presence of ischemia did not alter treatment effectiveness. In COURAGE, patients with stable coronary artery disease (CAD) received optimal medical therapy (OMT) with or without percutaneous coronary intervention (PCI). Of note, in the nuclear substudy of COURAGE, at least moderate ischemia at baseline was not associated with reduction of death or myocardial infarction (MI) from PCI added to OMT.

Moreover, a recent meta-analysis that included patients with stable CAD and ischemia documented by stress testing or fractional flow reserve and compared hard end points (death, nonfatal MI, unplanned revascularization, or angina) from PCI and OMT vs OMT alone did not show any benefits from PCI in this subset of patients with CAD.^6^ Consequently, whether the presence of ischemia documented during stress testing is associated with major cardiovascular events, regardless of the treatment applied, remains unproven.

To our knowledge, few studies have assessed the association of documented ischemia with long-term cardiovascular outcomes in patients with stable CAD, especially considering OMT. The ongoing National Heart, Lung, and Blood Institute–sponsored International Study of Comparative Health Effectiveness with Medical and Invasive Approaches (ISCHEMIA) trial^7^ is currently investigating this issue. In addition, no study evaluated the possible effects of chronic ischemia on the evolution of left ventricular function.

The second Medical, Angioplasty, or Surgery Study (MASS II) is a randomized clinical trial designed to compare the long-term effects of OMT, PCI, or coronary artery bypass graft (CABG) surgery in patients with stable multivessel CAD and preserved systolic ventricular function who are appropriate candidates for any of the 3 therapies.^8,9^ The present study is a post hoc analysis of MASS II to evaluate the association of stress-induced documented ischemia with the occurrence of major adverse cardiovascular events (MACEs) and the evolution of the left ventricular ejection fraction (LVEF) in patients with multivessel CAD and initially preserved ventricular function, 10 years after aggressive OMT with or without PCI or CABG.

## Methods

### Study Design

This was a prospective cohort study using data from the randomized clinical trial MASS II (isrctn.org identifier: ISRCTN66068876). Patients were enrolled in MASS II between May 1, 1995, and May 31, 2000; the present analysis used data collected at the 10-year follow-up.

### MASS II Patient Selection

Patients with angiographically documented, proximal multivessel coronary stenosis of more than 70% by visual assessment and documented ischemia were considered for inclusion. Ischemia was documented by either exercise stress testing (EST) or the typical stable angina assessment of the Canadian Cardiovascular Society (class II or III).^10^ Patients were enrolled and randomized if the surgeons, attending physicians, and interventional cardiologists agreed that revascularization could be attained by either PCI or CABG.^8,9^

The trial was approved by the ethics committee of the Heart Institute (InCor) of the University of São Paulo Medical School in São Paulo, Brazil, and all procedures were performed in accordance with the Declaration of Helsinki.^11^ Written informed consent was obtained from patients, who were randomly assigned to a treatment group.

The clinical criteria for exclusion were mandatory revascularization, left main coronary artery stenosis of 50% or more, ventricular aneurysm that required surgical repair, LVEF less than 40%, a history of PCI or CABG, congenital or valvular heart disease, or another coexisting condition that was a contraindication for CABG or PCI.

### Treatment Interventions

In MASS II, all patients were placed on an OMT until the end of the follow-up. Patients were randomized to either continue with aggressive OMT alone or undergo PCI or CABG in addition to OMT. The use of drugs with known cardiovascular benefits was adjusted throughout the study according to treatment guidelines. Patients were also encouraged to stop smoking, improve their dietary behaviors, and exercise regularly.

Investigators were required to perform optimum coronary revascularization. For patients assigned to PCI, the procedure was available within 3 weeks after randomization and was performed according to a standard protocol.^12^ Techniques used for catheter-based therapy included bare metal stent placement, laser angioplasty, directional atherectomy, and balloon angioplasty. For patients assigned to CABG, the procedures were available within 12 weeks after randomization, and revascularization was performed with standard surgical techniques^13^ using saphenous vein grafts, internal mammary arteries, and other arterial conduits. No off-pump CABG was performed.

### Follow-up

Adverse and other clinical events were tracked from randomization. Patients were evaluated and angina symptoms were graded,^10^ with follow-up visits every 6 months for at least 10 years at the Heart Institute. Patients underwent regular electrocardiography and routine blood tests.

Myocardial infarction was defined as the presence of significant new Q waves in at least 2 electrocardiogram leads or symptoms compatible with MI associated with creatine kinase MB fraction concentrations that were more than 3 times the upper limit of the reference range.

The predefined primary composite end point was the incidence of total mortality, Q-wave MI, or refractory angina that required revascularization.

### Exercise Stress Testing

A symptom-limited treadmill EST according to the modified Bruce Protocol^14^ was performed on participants in MASS II before randomization to one of the proposed treatments (OMT, PCI, or CABG) and thereafter every year until the end of the study unless contraindicated. We considered stress-induced ischemia as the presence of exertional angina or an ST-segment depression (horizontal or downsloping of 1 mm for men and 2 mm for women) at 0.08 second after the J point.

### LVEF Assessment

Participants in the MASS II trial underwent transthoracic echocardiography in 2 different periods: before randomization and after 10 years of follow-up. All echocardiographic factors assessed were predefined, and images were analyzed in a core laboratory by expert physicians.

Left ventricular ejection fraction was measured by the biplane method (also known as the Simpson method)^15^ when the endocardial border of the left ventricle was well defined and whenever regional wall-motion abnormalities were present or, alternatively, by the Teichholz method.^15^

### Statistical Analysis

Data analysis was performed from February 1, 2016, to April 1, 2017. Baseline characteristics were summarized for all patients as percentages for categorical variables and as means with SDs for continuous variables. Means were compared using the unpaired *t* test for parametric variables^16^ and the Mann-Whitney test for nonparametric variables. Tests were 2-sided. The homogeneity between proportions was evaluated using the χ^2^ or the Fisher exact test.^16^

The event-free survival time was defined as the interval between random assignment and the occurrence of the first of the components of a primary end point or the latest follow-up. Event-free survival was estimated by the Kaplan-Meier method, and differences among groups were assessed with the log-rank test.

Finally, multivariable Cox regression models were calculated to assess the relationship between ischemia and the primary composite end point by adjusting for sex, age, history of MI, number of diseased vessels, and initial CAD treatments.

All data were analyzed according to the intention-to-treat principle, and values of *P* < .05 were considered statistically significant. Statistical analysis was performed with SPSS, version 17.0 for Windows (IBM Corp).

## Results

### Baseline Clinical Characteristics

Between May 1, 1995, and May 31, 2000, 611 eligible patients who met all entry criteria were randomly assigned to 1 of 3 therapeutic strategies: PCI, OMT, or CABG. The vital status of all randomly assigned patients was determined on February 28, 2011. For patients still alive, the minimum length of follow-up was 10 years, and the maximum was 15 years (mean [SD], 11.4 [4.3] years). No patient was lost during follow-up.

In all, 535 patients were randomized. Of these, 373 (69.7%) were men, 162 (30.3%) were women, and the mean (SD) age for the entire cohort was 59.7 (9.2) years. Randomization created balanced treatment groups (176 in OMT, 180 in PCI, and 179 in CABG) with respect to important prognostic characteristics, except for the occurrence of previous MI (more frequent in the PCI group) and total cholesterol levels (higher in the PCI group; eTable in the Supplement).

Before randomization, 535 patients underwent the EST, among whom 270 (50.5%) had stress-induced ischemia whereas 265 (49.5%) did not. None of the characteristics were significantly different between the 2 groups, except for previous MI (more frequent in patients without ischemia) (Table 1). Treatment allocation was similar among those with and without ischemia (80 patients [30.0%] with stress-induced ischemia vs 95 [35.8%] without for OMT, 91 [33.7%] vs 89 [33.6%] for PCI, and 98 [36.3%] vs 81 [30.6%] for CABG, *P* = .26).

**Table 1.** Characteristics of the Population Stratified by Baseline Stress Test Results

Characteristic	Stress-Induced Ischemia (n = 270)^a^	No Stress-Induced Ischemia (n = 265)^a^	*P* Value
Demographic variables			
Age, mean (SD), y	59.6 (9.3)	59.6 (8.9)	.89
Age ≥65 y	79 (29.3)	94 (35.5)	.15
Male	193 (71.5)	180 (68.0)	.42
Female	77 (28.5)	85 (32.0)	.53
Clinical history and status			
Current or past smoker	89 (33.0)	95 (35.8)	.54
Hypertension	164 (60.7)	150 (56.6)	.38
Diabetes	95 (35.2)	103 (38.9)	.43
History of MI	102 (37.8)	131 (49.4)	.008
Laboratory values, mean (SD), mg/dL			
Total cholesterol	225.1 (46.8)	222.5 (47.8)	.32
LDL cholesterol	150.7 (41.3)	145.3 (42.5)	.17
HDL cholesterol	38.2 (10.4)	36.7 (10.2)	.04
Triglycerides	186.8 (102.0)	198.4 (115.2)	.34
Angiographic profile			
Double-vessel disease	113 (41.8)	111 (41.9)	>.99
Triple-vessel disease	157 (58.1)	154 (58.1)	
Proximal LAD disease	243 (90.0)	238 (89.8)	
CAD treatment			
OMT	81 (30.0)	95 (35.8)	.26
PCI	91 (33.7)	89 (33.6)	
CABG	98 (36.3)	81 (30.6)	

Abbreviations: CABG, coronary artery bypass graft; CAD, coronary artery disease; HDL, high-density lipoprotein; LAD, left anterior descending coronary artery; LDL, low-density lipoprotein; MI, myocardial infarction; OMT, optimal medical therapy; PCI, percutaneous coronary intervention.

SI conversion factors: To convert total, LDL, and HDL cholesterol to millimoles per liter, multiply by 0.0259; triglycerides to millimoles per liter, multiply by 0.0113.

^a^ Values are expressed as number (percentage) unless otherwise indicated.

In addition, 472 patients (88.2%) had a history of exertional angina and 63 (11.8%) did not. Of the 472 patients with angina symptoms, 246 (52.1%) had positive EST results, 88 (18.6%) had negative results, and 138 (29.2%) had inconclusive results. Of the 63 patients without any angina symptoms, 24 (38.1%) had positive EST results, 14 (22.2%) had negative results, and 25 (39.7%) had inconclusive results.

### Exercise Stress Testing Analysis

The overall major cardiac events at the 10-year follow-up of patients stratified by the results of the baseline stress tests are provided in Table 2. No association was found between the presence of ischemia at baseline and the occurrence of MACE.

**Table 2.** Major Adverse Cardiac Events at 10 Years Stratified by Baseline Stress Test Results

Outcomes	Stress-Induced Ischemia (n = 270)^a^	No Stress-Induced Ischemia (n = 265)^a^	Hazard Ratio (95% CI)	*P* Value^b^
Overall mortality	80 (29.6)	84 (31.7)	0.80 (0.58-1.11)	.18
Acute myocardial infarction	33 (12.2)	41 (15.5)	0.85 (0.54-1.36)	.51
Additional intervention	87 (32.2)	68 (25.7)	1.26 (0.92-1.74)	.15
Primary end point^c^	153 (56.7)	145 (54.7)	1.00 (0.80-1.27)	.95

^a^ Values are expressed as number (percentage) unless otherwise indicated.

^b^ Cox proportional hazards models adjusted for sex, age, 2- or 3-vessel coronary artery disease, coronary artery disease treatments, and previous myocardial infarction.

^c^ The primary end point was the occurrence of the first clinical event (acute myocardial infarction, additional intervention, or overall mortality).

**Figure 1.**
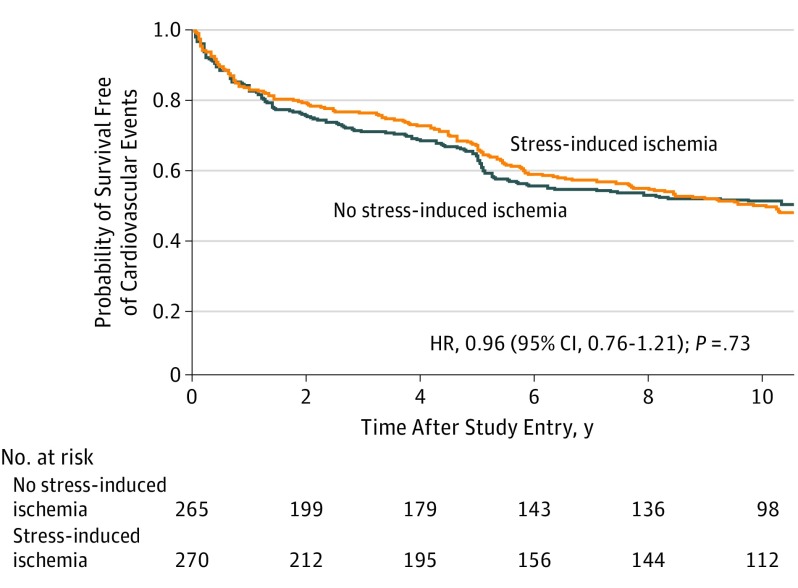
Kaplan-Meier Curves for Survival Free of Cardiovascular Events According to Stress-Induced Ischemic Status at Baseline HR indicates hazard ratio.

Figure 1 shows the Kaplan-Meier survival curves of the occurrence of cardiovascular events in patients stratified by the presence of stress-induced ischemia. No significant differences were found between patients with or without ischemia regarding survival free of the combined cardiovascular end points (hazard ratio [HR], 0.96; 95% CI, 0.76-1.21; *P* = .73). After adjusting for baseline prognostic variables (sex, age, previous MI, number of diseased vessels, and initial CAD treatments), the presence of stress-induced ischemia was not found to be associated with the occurrence of cardiovascular events (HR, 1.00; 95% CI, 0.80-1.27; *P* = .95).

In addition, among patients with and without stress-induced ischemia, survival free of cardiovascular events was more adverse in OMT and PCI groups, compared to that in the CABG group (eFigure 1 in the Supplement). The pairwise treatment comparisons of the occurrence of the primary composite end point demonstrated no differences between the OMT and PCI groups in patients with (HR, 0.81; 95% CI, 0.55-1.18; *P* = .27) and without (HR, 0.87; 95% CI, 0.60-1.28; *P* = .48) stress-induced ischemia.

### LVEF Assessment

**Figure 2.**
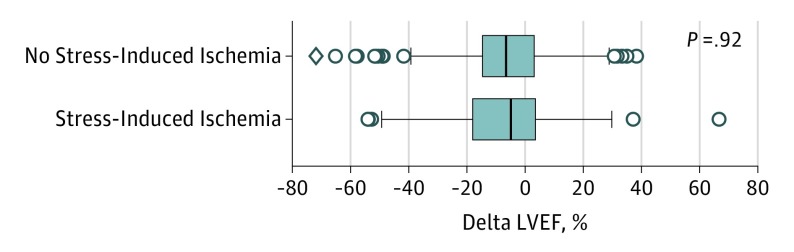
Changes in Left Ventricular Ejection Fraction (LVEF) According to the Presence of Stress-Induced Ischemia The delta LVEF (difference in reduction of LVEF) is calculated as 100 × [(LVEF at 10 years − LVEF at baseline)/LVEF at baseline]. The vertical line in the middle of each box indicates the median; left and right borders of the box, the interquartile ranges; whiskers, the maximum and minimum values excluding the outliers; and data points beyond the whiskers, outliers.

Of the 535 patients who underwent EST, 320 had their ventricular function assessed with echocardiography at baseline and after the 10-year follow-up. The echocardiographic assessment identified similarities in LVEF evolution among patients with and without stress-induced ischemia. Irrespective of the ischemic status, both groups exhibited a slight decline in LVEF, which is represented by the difference in reduction (median [SD], −4.9% [18.7%] vs −6.6% [20.0%], respectively, for groups with and without ischemia; *P* = .97) (Figure 2).

The outcomes of the different treatment strategies were analyzed in the subgroups of patients with and without stress-induced ischemia. In both conditions, regardless of the treatment applied, patients experienced the same pattern and magnitude of ventricular function variation, with a slight decline in LVEF represented by the difference in reduction for those without stress-induced ischemia (eFigure 2 in the Supplement).

## Discussion

This study evaluated the occurrence of cardiovascular events and changes in left ventricular function in patients (participants in the MASS II randomized clinical trial) with multivessel CAD who underwent 1 of 3 therapeutic strategies (alone or with PCI or CABG) according to the presence or absence of exercise stress-induced ischemia at baseline. The results show that the presence or absence of documented myocardial ischemia appears to have no association with long-term cardiovascular outcomes and changes in LVEF in patients with stable multivessel CAD and preserved ventricular function.

Results of previous trials^9,17,18,19^ presumed the presence of myocardial ischemia based on symptoms, ischemic test results, and coronary anatomy findings. Their findings suggest that myocardial ischemia might not play a role as an additional risk factor for events in patients with stable CAD irrespective of the treatment applied. However, the influence of objective, documented ischemia on treatment outcomes is not well established.

One of the first studies that assessed the comparative results of CAD treatments in patients with documented ischemia was conducted by Hachamovitch et al.^20^ In their analysis of registry data, the authors compared OMT with revascularization strategies in patients who underwent myocardial perfusion stress tests. Their findings showed that patients with no or mild ischemia in the OMT group had a survival benefit, whereas those with moderate to severe ischemia had better survival outcomes if they were revascularized. Despite the interesting results, major methodological concerns, such as the differences in baseline variables between groups, no defined medical therapy, the lack of an indication for revascularization procedures, and selection bias, compromised the interpretation of the study findings.

On the other hand, contemporary randomized studies have shown different results regarding the association of ischemia with outcomes and treatment effects. The COURAGE nuclear substudy^21^ performed serial rest/stress myocardial perfusion scintigraphy before treatment and 6 to 18 months after randomization in 314 of the 2287 participants in the original trial. The authors observed that the reduction in ischemia achieved more frequently in PCI added to the OMT group was not associated with a lower risk of death or MI in the adjusted analysis. Another substudy of the COURAGE trial including 1381 participants found similar event rates with both treatments irrespective of the extent and severity of ischemia at baseline.^5^ Therefore, results from the COURAGE trial are similar to those of the present analysis.

In the Fractional Flow Reserve vs Angiography for Multivessel Evaluation (FAME) 2 trial,^22^ which studied patients with functionally significant stenosis, as determined by a measurement of fractional flow reserve less than 0.80, the addition of PCI to OMT was evaluated for the prevention of the primary composite end point (death, MI, or urgent revascularization). The researchers found that fractional flow reserve–guided PCI plus OMT, compared with OMT alone, was associated less frequently with the primary composite end point. However, contributing to the result was the decreased need for urgent revascularization in the PCI group. In addition, the unblinded nature of FAME 2 may have biased the results in favor of more urgent revascularizations in patients randomized to OMT.

Of note, in the subset of patients with left ventricular dysfunction, a substudy of the Surgical Treatment for Ischemic Heart Failure (STICH) trial also demonstrated that inducible myocardial ischemia did not identify patients with worse prognosis or those with greater benefit from CABG over OMT.^23^ The STICH trial randomized patients with CAD and an ejection fraction less than or equal to 35% to CABG or OMT.

Supporting the findings of these later studies, a meta-analysis of contemporary trials compared the clinical outcomes of PCI and OMT vs those of OMT alone exclusively in patients with stable CAD and documented myocardial ischemia.^6^ The authors concluded that, in patients with documented ischemia, PCI was not able to reduce cardiovascular events.

A prior study of the MASS group among patients with multivessel CAD, evaluated the evolution of LVEF after 10 years of follow-up^24^ and found no difference among the 3 treatment groups. Also, the subgroup of patients with and without stress-induced ischemia demonstrated by EST at the end of follow-up had the same evolution of ventricular function. In the present study, the presence of ischemia in EST at baseline was not associated with worse evolution of LVEF.

Although the present study did not have data about high-risk findings in EST, more than 50% of the studied patients had triple-vessel disease and almost 90% had proximal left anterior descending coronary artery involvement, which characterize a higher-risk population. Despite this high risk, the results of this study suggest that, in patients with stable CAD, documented ischemia observed by ischemic changes during EST was not associated with different 10-year cardiovascular outcomes and worsening of ventricular function compared with that in patients with nonischemic EST.

Although the presence of documented ischemia has been identified as a possible marker of a higher-risk population and an indication for myocardial revascularization procedures to protect the myocardium from the chronic, deleterious effects of ischemia over time, the present study’s findings do not support this assumption. The delicate imbalance between oxygen supply and demand at stress is a consequence and does not seem to be a factor for impairment of ventricular function or coronary events during a long-term follow-up.

A possible physiopathological explanation for this finding could be that the functional information about myocardial stress-induced ischemia may not indicate atherosclerotic plaque instability, which is the major factor responsible for clinical events. Since myocardial ischemia is associated with the degree of stenosis and atherosclerotic burden, we must consider that its presence, even if significant, may remain stable for a long time while the plaque stability persists. Thus, the results of such functional tests should be interpreted cautiously, and not isolated from other clinical information when revascularization strategies are being considered for the treatment of CAD patients. The results from the ongoing ISCHEMIA trial^7^ are expected to introduce new information regarding this subject.

### Limitations and Strengths

This study has limitations. First, because this was a retrospective analysis, all aspects covered are related to this type of study. However, all data were derived from a randomized clinical trial, with predictors and outcome variables collected prospectively in a detailed database. Second, medical therapy changed during the evolution of the trial, which may have influenced the findings; such evolution is inherent in long-term follow-up studies. The changes noted occurred in all study patients, irrespective of the group they were placed in later. Last, this was a single-center study, which may limit the external validation of the analysis. However, the homogeneity of medical conduct, medical therapy, and indications for coronary interventions, especially during the long-term follow-up, reduce the limitations of the present study.

## Conclusions

In this study, regardless of the therapeutic strategy applied, the presence of documented ischemia did not appear to be associated with the occurrence of major adverse events or changes in left ventricular function among the participants selected from the MASS II trial.

## Notes

Supplement. **eTable.** Baseline Characteristics of the 535 Patients With Exercise Stress Tests According to Treatment in MASS II Trial **eFigure 1.** Survival Free of Cardiovascular Events According to Initial Assigned Treatment in Patients With (1A) and Without (1B) Stress Induced Ischemia **eFigure 2.** Changes in Left Ventricular Ejection Fraction According to the Presence or Absence of Myocardial Ischemia in Each Treatment Group After 10-Year Follow-up
