# Supplementary material for: Association Between Stress Testing–Induced Myocardial Ischemia and Clinical Events in Patients With Multivessel Coronary Artery Disease
Source: JAMA Intern Med. 2019 Jul 22;179(10):1345–51. doi: 10.1001/jamainternmed.2019.2227 (PMC6647357; doi:10.1001/jamainternmed.2019.2227)
Supplement: Supplement. — eTable. Baseline Characteristics of the 535 Patients With Exercise Stress Tests According to Treatment in MASS II Trial eFigure 1. Survival Free of Cardiovascular Events According to Initial Assigned Treatment in Patients With (1A) and Without (1B) Stress Induced Ischemia eFigure 2. Changes in Left Ventricular Ejection Fraction According to the Presence or Absence of Myocardial Ischemia in Each Treatment Group After 10-Year Follow-up [file jamainternmed-179-1345-s001.pdf]

Supplementary Online Content

Garzillo CL, Hueb W, Gersh B, et al. Association between stress testing–induced myocardial ischemia and clinical events in patients with multivessel coronary artery disease. *JAMA Intern Med*. Published online July 22, 2019. doi:10.1001/jamainternmed.2019.2227

eTable. Baseline Characteristics of the 535 Patients With Exercise Stress Tests According to Treatment in MASS II Trial

eFigure 1. Survival Free of Cardiovascular Events According to Initial Assigned Treatment in Patients With (1A) and Without (1B) Stress Induced Ischemia

eFigure 2. Changes in Left Ventricular Ejection Fraction According to the Presence or Absence of Myocardial Ischemia in Each Treatment Group After 10-Year Follow-up

This supplementary material has been provided by the authors to give readers additional information about their work.

© 2019 Garzillo CL et al et al. *JAMA Internal Medicine.*

eTable. Baseline Characteristics of the 535 Patients With Exercise Stress Tests According to Treatment in MASS II Trial

	PCI (N=180)	CABG (N=179)	MT (N=176)	*P*-value
**Demographic variables**				
Age, mean (SD), y	59.5 (9.4)	59.5 (8.7)	59.9 (9.3)	0.91
Age $\geq$ 65 years (%)	32	31	33	0.90
Male (%)	66	73	70	0.24
Current or past smoker (%)	31	35	36	0.62
**Clinical History and Status**				
History of MI (%)	50	42	38	0.04
History of hypertension (%)	60	62	53	0.21
Treated diabetes mellitus (%)	31	39	40	0.14
**Laboratory values**				
Total Cholesterol, mean (SD)	230.1 (51.2)	217.7 (47.4)	223.6 (42.1)	0.048
LDL cholesterol, mean (SD)	151.0 (44.9)	144.1 (43.0)	149.1 (37.2)	0.27
HDL cholesterol, mean (SD)	36.8 (9.5)	37.8 (10.7)	37.9 (10.8)	0.56
Triglycerides, mean (SD)	205.9 (115.1)	182.3 (102.9)	189.2 (10.7)	0.11
**Positive treadmill test (%)**	50	54	46	0.26
**Angiographic profile**				
Double-vessel disease (%)	41	44	41	0.85
Triple vessel disease (%)	59	56	59	
Proximal LAD disease (%)	89	92	88	0.42

PCI, percutaneous coronary intervention; CABG, coronary artery bypass graft; MT, medical therapy; LDL, low-density lipoprotein; HDL, high-density lipoprotein; LAD,

© 2019 Garzillo CL et al et al. *JAMA Internal Medicine.*

left anterior descending coronary artery. Unless otherwise indicated, data are mean±SD. Laboratory values are expressed in mg/dL.

© 2019 Garzillo CL et al et al. *JAMA Internal Medicine.*

eFigure 1. Survival Free of Cardiovascular Events According to Initial Assigned Treatment in Patients With (1A) and Without (1B) Stress Induced Ischemia

Figure 1A

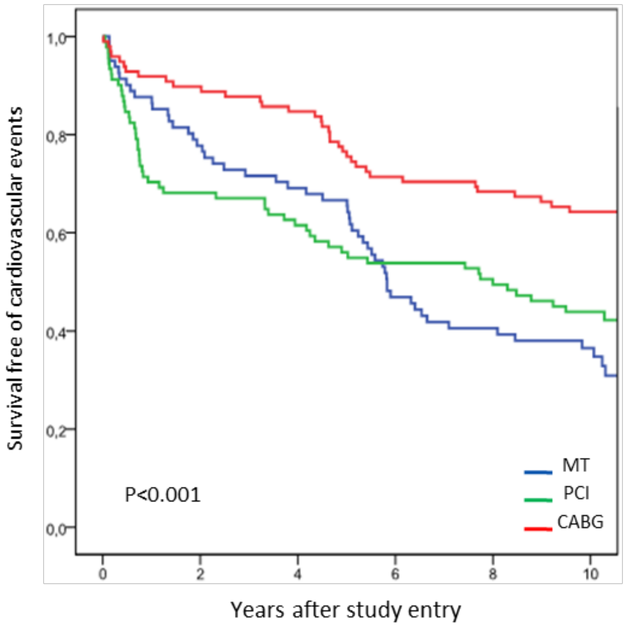

	initial	2 y.	4 y.	6 y.	8 y.	10 y.
MT	81	62	56	38	31	22
PCI	91	61	55	49	45	31
CABG	98	87	82	69	66	57

Figure 1B

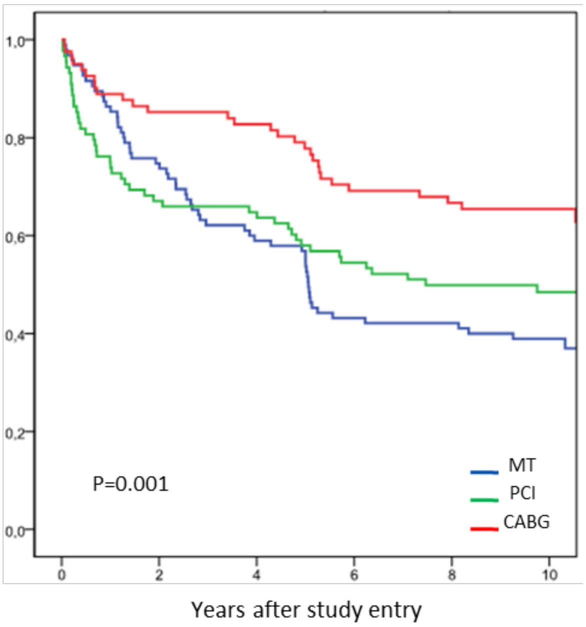

	initial	2 y.	4 y.	6 y.	8 y.	10 y.
MT	95	70	56	40	39	26
PCI	88	58	56	46	43	31
CABG	81	69	66	56	53	39

MT, medical treatment; PCI, percutaneous coronary intervention; CABG, coronary artery bypass graft. The primary endpoint is the sum of the first clinical event (AMI, additional interventions and overall mortality).

© 2019 Garzillo CL et al et al. *JAMA Internal Medicine.*

eFigure 2. Changes in Left Ventricular Ejection Fraction According to the Presence or Absence of Myocardial Ischemia in Each Treatment Group After 10-Year Follow-up

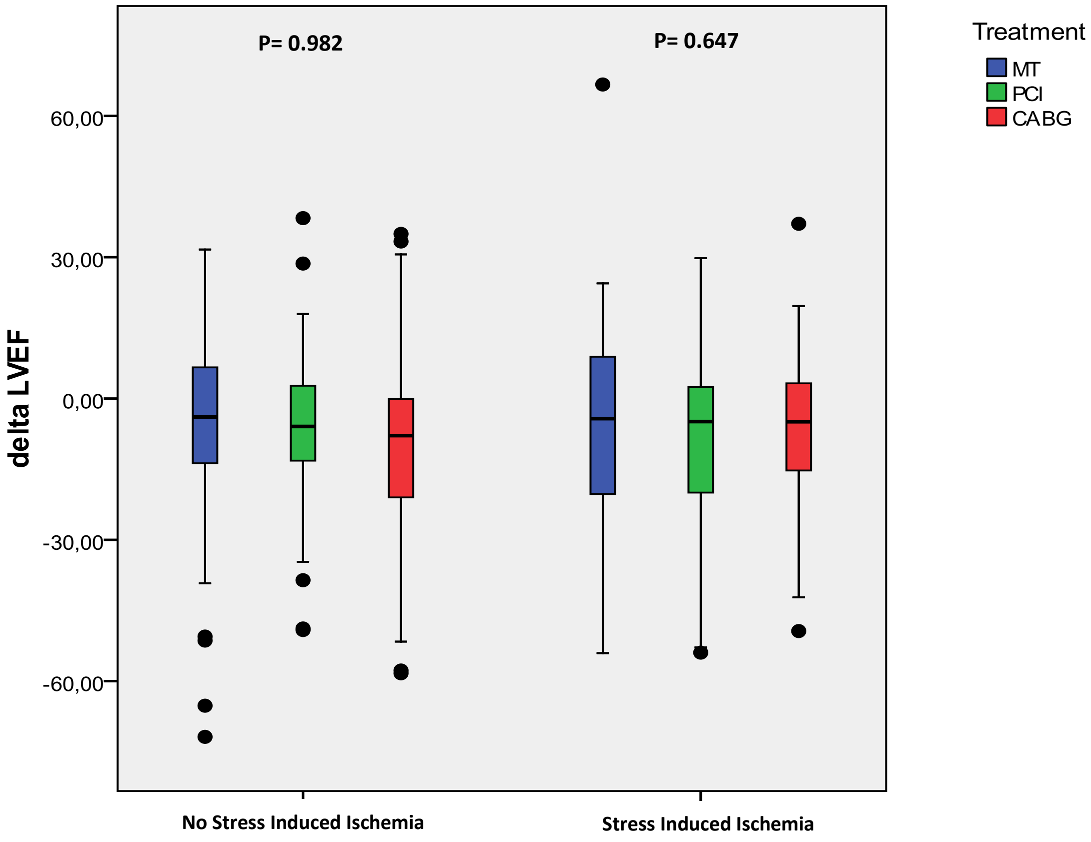

MT: medical therapy; PCI: percutaneous coronary intervention; CABG: coronary artery bypass graft; delta LVEF: 100 X (LVEF 10 year– LVEF baseline)/ LVEF baseline; values expressed in median ± SD.

© 2019 Garzillo CL et al et al. *JAMA Internal Medicine.*
